# Efficient ultrafast laser writing with appropriate polarization

**DOI:** 10.1038/s41377-023-01161-y

**Published:** 2023-05-08

**Authors:** Xiujian Li, Wenke Xie

**Affiliations:** 1 College of Sciences, National University of Defense Technology, Changsha, 410073 China; 2 Tiansun National Lab, Changsha, 410073 China; 3 School of Physics and Electronics, Central South University, Changsha, 410083 China

**Keywords:** Optical data storage, Nonlinear optics

## Abstract

Appropriate polarization utilization makes the electric field vector direction and the statistically oriented localized states suitable for enhancing light-matter interactions so as to improve the efficiency of ultrafast laser writing, which will remarkably reduce the pulse energy and increase the processing speed for high density optical data storage, as well as manufacturing three-dimensional integrated optics and geometric phase optical elements.

Laser writing, which has been considered to be a prospective technology and have promising applications in three-dimensional manufacturing field and information processing field since rapid crystallization and equally rapid devitrification of amorphous chalcogenides have been observed when they are exposed to short laser pulses in 1970s^1,2^. With the fundamental physical interpretation of Q-switching and mode-locking to produce ultrashort laser pulses presented in 1971^3^, Donna Strickland and Gérard Mourou proposed the method to achieve ultrashort laser pulses^4^ in 1985 and shared the 2018 Nobel Physics Prize, which greatly promote the development of ultrafast lasers and the corresponding applications such as ultrafast laser writing.

With ultrahigh peak power for enhancing the light-matter interactions in a ultrashort time without collateral damage of the material, and even only change in a very tiny focusing region, ultrafast laser writing has good applications in three-dimensional integrated optics^5–9^, direct printing of optical elements with nano-structures^10–16^ and high density optical data storage^17,18^. Surely, efficiency and resolution should be always the major keys for ultrafast laser writing.

In the Big Data Era right now, we unprecedentedly need better reliable high-intensity storage methods besides storage tape, magnetic disk and solid state disk, optical data storage is beyond question, as multiplexed data storage techniques can provide ultra-broadband I/O which is essential for daily data management and many data processing systems such as optical computing systems^19,20^. We know that, the silica glass plays a very important role in optical storage, for instance, the 5D optical data storage based on direct writing in silica glass can offer high data capacity up to 20TB per 5-inch disk and virtually unlimited lifetime^21^. How to achieve remarkable artificial optical anisotropy or birefringence without stronger light absorption, which indicates efficient light-matter interaction, is still an important issue.

People are deepening researches into the physical mechanism and dynamics of the light-matter interactions to find solutions. Herein, writing in this issue of Light: Science & Applications, Yuhao Lei and colleagues at University of Southampton, University of Latvia and University of Eastern Finland report for the first time a method for efficient creation of anisotropic nanopores and related birefringence in silica glass using elliptically polarized laser pulses^22^. Based on their previous work on 5D optical data storage technique, in which the background between voxels was removed by an algorithm for the precise retardance measurement^17^, the authors achieve ultrafast laser direct writing with elliptical polarization in silica glass, and demonstrate that the nonlinear absorption is about 2.5 times weaker while results in form birefringence about twice that of linearly polarized light, i.e., the maximum induced birefringence occurs at an ellipticity of 0.6 and not at linear polarization, surely beyond intuition which figures that artificial optical anisotropy to be the strongest when created by light with linear polarization. They find that, an obvious increase in retardance with increasing ellipticity of light is a property of the type X modification, which suggests a peculiar physical mechanism for the formation of anisotropic nanopores that is different from the self-organized mechanism of nanogratings formation.

In order to reveal the physical mechanism of the birefringent modification, the authors perform additional nonlinear absorption measurements and annealing experiments. Associated with the birefringence and SEM image of the laser writing region, they confirm that, as an elliptically polarized pulse can be considered as a combination of linear and circular polarized components, the birefringent modification by elliptical polarization is a consequence of the balance between the concentration of nanopores with a more efficient formation at circular polarization and their shaping due to the anisotropy of the near-field enhancement produced by the linear polarization component. Basically, from a dynamic perspective, the circularly polarized light with its rotating electric field direction can access a larger part of the statistically oriented localized states compared to linearly polarized light, and the increase in birefringence with elliptical polarization should be mainly due to the form birefringence and not to stressinduced birefringence. The authors also performed digital document laser direct writing in 50 layers silica glass as a demonstration of elliptical polarization writing, in which two levels of retardance were obtained with two different light ellipticity values (0.6 and 0.8) with an energy of 215 nJ and a pulse number of 20 (while 30 for linearly polarized pulses), meaning that 4 bits of information can be encoded into one voxel of 5D optical data storage and achieve data capacity beyond 350 GB in a 5-inch disc with ultrahigh readout accuracy. With multi-channel parallel recording, the write speed for elliptical polarization can be improved up to more than 2.5 MB/s at a laser repetition rate of 5 MHz or higher.

A scheme of ultrafast laser writing with polarization and vortex selections for optical data storage is shown in Fig. 1. Successful efficient ultrafast laser writing in silica glass with elliptical polarization in the presented work demonstrates that, it is promising to reduce the pulse energy and increase the recording speed in high density optical data storage, as well as reduce the manufacturing time of three-dimensional integrated optics and geometric phase optical elements. Furthermore, as the laser writing efficiency in some materials heavily depend on the transient electric field vector direction as well as the statistically oriented localized states, besides the linearly polarization and the elliptically polarization beams, the vector beams (the radially polarization and the azimuthally polarization)^23,24^, and even the vortex beams^25,26^ can be further considered for laser writing efficiency improvements.

**Fig. 1:**
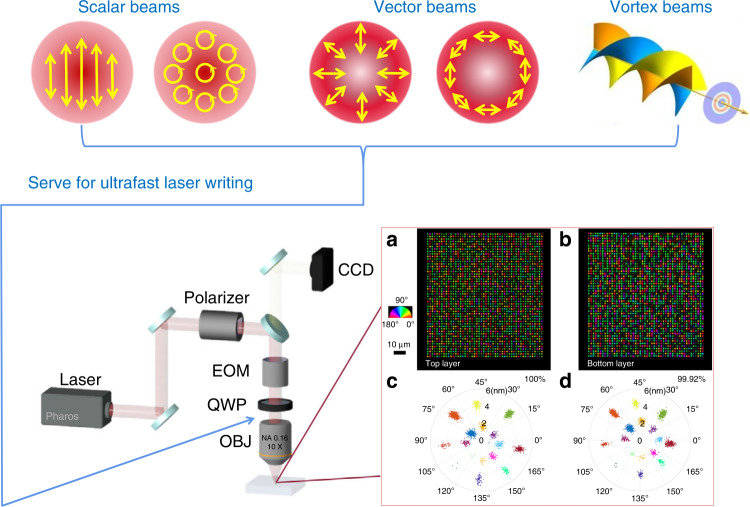
Scheme of ultrafast laser writing with polarization and vortex selections for optical data storage. **a**, **b** The birefringence image of data voxels from the top and bottom layer. **c**, **d** Polar diagram of the measured retardance and azimuth of all voxels in (**a**) and (**b**)
